# Evaluating the reasons for nonattendance to outpatient consultations: is waiting time an important factor?

**DOI:** 10.1186/s12913-022-08033-y

**Published:** 2022-05-09

**Authors:** Bernadeta Zykienė, Vytenis Kalibatas

**Affiliations:** Faculty of Public Health, Medical Academy, Lithuanian University of Health Sciences, Tilžės str. 18, Kaunas, Lithuania

**Keywords:** Nonattendance, Reasons for nonattendance, Waiting time, Outpatient, Consultation

## Abstract

**Background:**

Nonattendance is a common problem worldwide. Important factors for nonattendance are a queue or the waiting time until the planned service.

**Aims:**

The aims of this study were to identify the reasons for nonattendance to planned consultations, assess the waiting time from registration to access to an outpatient specialist consultation, and identify the associations between the reasons for nonattendance and the waiting time until the planned outpatient specialist consultation.

**Methods:**

A cross-sectional study based on a phone questionnaire was conducted among patients not attending a planned consultation at the outpatient department of the Lithuanian University of Health Sciences Kaunas Hospital in Kaunas, Lithuania. A total of 972 phone calls were made, and 389 telephone surveys were completed.

**Results:**

The mean respondents’ waiting time until the planned outpatient consultation was 15.13 ± 10 days. The highest proportion of nonattendance was observed when the wait time was between 6 and 17 days.

More often, the patients did not attend the planned outpatient consultation due to worsened health status (24.69%), unidentified personal problems (14.91%), work-related problems (13.62%) and being unaware about the appointment (11.82%). A longer waiting time was significantly associated with the following reasons for nonattendance: work-related problems, health problems solved at another health care institution, unidentified personal problems and unknown reasons for nonattendance. The highest proportions of nonattending patients had consultations registered with neurologists (17.0%), traumatologists (11.3%) and cardiologists (10.5%).

**Conclusions:**

Patients did not identify the long waiting time until outpatient specialist consultation among the main reasons for nonattendance. The issue of waiting time is not an important aspect of nonattendance.

## Introduction

The issue of nonattendance to a planned specialist consultation is a common problem worldwide. Foreknowledge of nonattendance to consultations can reduce health care costs and improve the quality and efficiency of health care. Medical institutions are exposed to financial losses, prolonged waiting times between registration and access to a specialist, and increased patient dissatisfaction with health care services due to registered patients failing to attend planned consultations. In the UK, one nonattendance costs approximately 160 pounds [1]. In the United States, it is estimated that the cost for nonattendance to appointments with physicians is 150 billion dollars per year [2]. Having assessed the possible factors associated with the risk for nonattendance, it is possible to create the necessary interventions to reduce the rate for nonattendance in medical institutions [3]. Waiting time is negatively associated with patient satisfaction [4] and is shown as a result of the higher incidence of nonattendance [5]. Thus, both nonattendance and a long waiting time until the planned outpatient consultation are common negative aspects in health care, but the association between them has received less attention.

The National Audit Office of Lithuania in the audit report “The Accessibility of Health Care Services and the Orientation Towards the Patient” stated that according to the opinion of the Lithuanian population, the main problem of health care in Lithuania is waiting too long until the outpatient specialized consultation (55% of respondents). It was found that only 11 of 71 specialized outpatient health care services were provided faster than within 31 days. Moreover, as a way of addressing the service accessibility issue, 17 percent of patients use paid services, and half of them do so because of long waiting times [6]. Outpatient waiting time is defined as the number of days that a patient has to wait from the time of registration to the appointment with the specialist (outpatient specialist consultation) following a general practitioner referral in Lithuania.

Previous studies provide contradictory results on the issues of waiting time and nonattendance. Some authors conclude that increased waiting is associated with a higher rate of nonattendance, while other authors have found the opposite. Cohen A stated that as waiting times increase, the frequency of nonattendance increases. The author concluded that with a waiting time of 1 to 7 days, the nonattendance rate is 21.2%; with a waiting time of 8 to 14 days, the nonattendance rate is 32.5%; and waiting for more than 15 days increases the nonattendance rate to 43.5% [7]. Bush R stated that an increased waiting time for a consultation is significantly related to the frequency of nonattendance. With a waiting time of 15 to 28 days, the odds ratio for nonattendance was 1.24; for a waiting time of 29 days or more, the odds ratio for nonattendance was 1.7 [8]. A study by Mohammadi I et al. found the opposite trend, with the highest number of nonattending patients waiting for a consultation for two weeks (35.4%) and 20.7% not waiting for more than two weeks [9].

There are a number of reasons why patients miss appointments, such as forgetfulness, a lack of information, illness (patient, family member), personal circumstances, other obligations, improvement of symptoms, an appointment for a further visit that the patient no longer needs, hospitalization, sleep, an inability to leave work, transportation, an appointment for a visit with a doctor other than the doctor the patient wanted, climatic conditions, family responsibilities, an inconvenient appointment time, appointment location and administrative issues [10–13]. Some of the reasons are related to factors that cannot be changed by treatment facilities (e.g., weather conditions). Typically, at least four of the reasons (“I forgot”, “I cancelled the visit”, “I arrived”, and “I didn’t know about the upcoming visit”) are reasons that health care institutions can change through certain measures and actions to reduce nonattendance [14]. The rate of nonattendance is associated with patients’ sociodemographic characteristics, waiting times, type of specialist and causes of nonattendance [15].

Considering the importance of this issue, the present study aimed to assess the waiting time from registration to access to an outpatient specialist consultation, identify the reasons for nonattendance to the planned consultation, and determine the associations between the reasons for nonattendance and waiting time until the planned outpatient specialist consultation. The hypotheses of this study were that one of the prevailing reasons for nonattendance is a waiting time that is too long until the planned outpatient specialist consultation (first hypothesis) and that a longer time between referral and appointment is associated with higher rates of nonattendance (second hypothesis). To the authors’ knowledge, no research on the waiting times and the rate of nonattendance in Lithuania has been previously published.

## Methods

### Settings

This study was conducted at the outpatient department of the Lithuanian University of Health Sciences Kaunas Hospital in Lithuania (Kaunas Hospital). Kaunas Hospital provides secondary outpatient and inpatient personal health care services. Planned outpatient health care services are provided by the outpatient department. Physicians with 27 different specialties provide consultations for outpatients. The planned consultations do not involve any diagnostic services. In 2018, 78,707 patients registered for planned specialist consultations at the outpatient department of Kaunas Hospital.

### Study design

A cross-sectional study based on a phone questionnaire was carried out among patients who did not attend planned outpatient consultations at Kaunas Hospital from January 1 to December 31, 2018. Patients older than 18 years who registered for a planned outpatient consultation and did not attend the consultation were included in the study.

To identify the reasons for nonattendance, a phone interview was conducted. Nonattending patients were questioned within 4–8 days after nonattendance. During the phone conversation, oral informed consent was obtained. After obtaining oral consent, a written consent form was completed on the participant’s behalf. During the phone questionnaire interview, data on respondents' sociodemographic characteristics, such as education, occupation, marital status and place of residence, were obtained. During the phone conversation, the respondents were asked to identify the reason(s) for nonattendance (an open question), if the problem the patient registered had been solved, and if the patient informed Kaunas Hospital about their nonattendance in advance.

Data on appointments missed in the previous week were collected from the Hospital Information System at the reception desk on Mondays. The following data were collected: name, sex, year of birth, place of residence, phone contact, time of the planned consultation (year, month, day, hour, minute), time of check-in for the consultation (year, month, day), physician specialty, and type of outpatient consultation (new or follow-up).

A phone questionnaire interview was performed every week on Tuesdays. Patients who did not attend the outpatient consultation in the previous week were sorted by the time of arrival in the queue and were given a number on the list. Patients were selected for a call using a real random number generator (www.random.org). If the patient did not answer the phone call, the call was not repeated. The next person was then selected randomly.

Statistical analysis was performed using SPSS 20.0. Means, proportions, and standard deviations were calculated for the descriptive statistics. The ANOVA *P* value was applied to determine whether there were any statistically significant differences between the means of independent (unrelated) groups. The Bonferroni Z test was used to determine if there was a significant difference between the means of two groups. Multivariable logistic regression analyses were performed to analyse the relationship between the reasons for nonattendance and waiting time until the planned outpatient consultation, as well as patient sex, age and appointment type. *P* values less than 0.05 were considered to be statistically significant.

## Results

A total of 3537 patients did not attend the planned consultations. The rate of nonattendance was 4.49%.

**Fig. 1:**
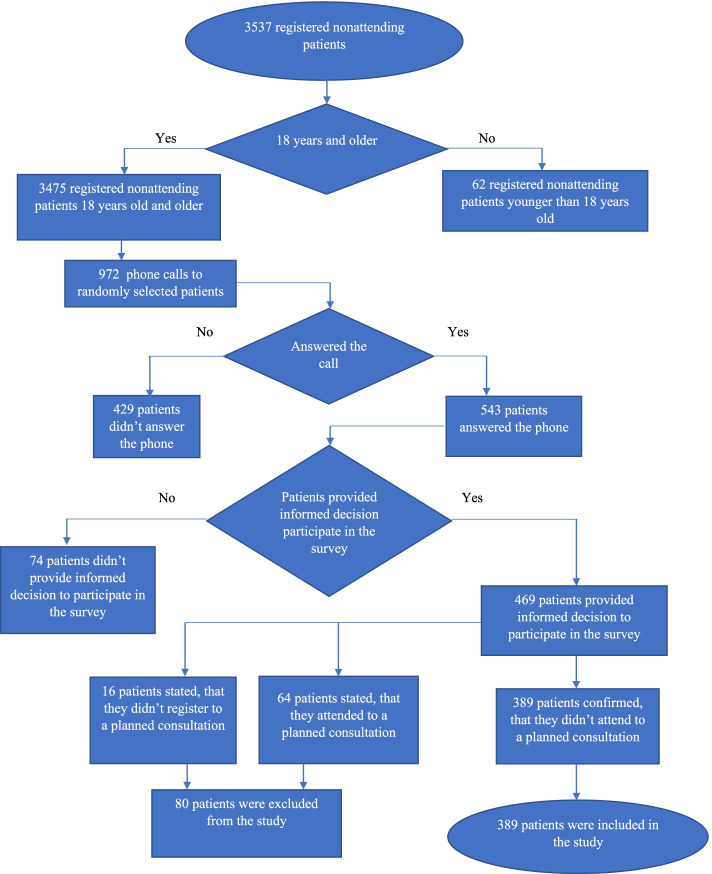
Participants in the study

There were 3475 adult (18 years and older) patients who did not attend the planned outpatient consultations. The required sample size was calculated using a sample size calculator. The confidence level was 95%, the population size was 3475, and the margin of error was 5%; therefore, the calculated sample size was 346. With the prediction that not all patients would consent to participate in the study, a total of 972 phone calls were made. Of these, 429 (44.14%) patients did not respond, 74 (7.41%) patients did not consent to participate in the study, 16 (1.65%) patients indicated that they did not register for an outpatient consultation at Kaunas Hospital, and 64 (6.58%) patients stated that they did attend the planned consultation. Therefore, 389 respondents' questionnaire data were used for the analysis (Fig. 1). The response rate was 40.02%.

The sociodemographic characteristics of the respondents are presented in Table 1.

**Table 1:** The sociodemographic characteristics of the respondents

Sociodemographic characteristics	Patients (*n* = 389)	Proportion (%)
Sex		
Females	247	63.5
Males	142	36.5
Age groups		
18–29 years old	60	15.4
30–49 years old	113	29.0
50–69 years old	152	39.1
70 years and older	64	16.5
Education		
Secondary school	110	28.3
Higher Education	124	31.9
Advanced Education	155	39.8
Marital status		
Married	239	61.4
Single	70	18.0
Divorced	42	10.8
Widowed	38	9.8
Occupation		
Employed	247	63.5
Unemployed	16	4.1
Retired person	101	26.0
Student	25	6.4
Residence		
Kaunas city	224	57.6
Other	165	42.4
Appointment type		
New (primary) referral	321	82.5
Follow-up (secondary) referral	68	17.5

Women did not attend the planned visits more often than men. The highest proportion of nonattending patients were in the age group of 50–69 (39.1%) years. A total of 61.4% of the respondents were married, and 63.50% were employed. More than half of the nonattending patients (57.6%) were living in Kaunas city (where a general hospital is located). A total of 82.5% of the patients were registered as a “new patient” at the primary consultation.

**Fig. 2:**
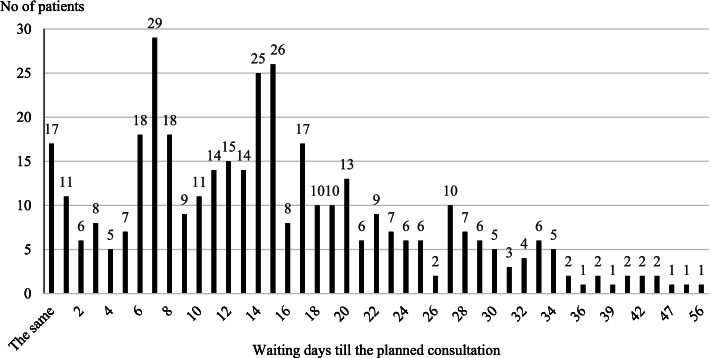
The distribution of the respondents according to their waiting time

The average respondents’ waiting time until the planned outpatient consultation was 15.13 ± 10 days (ranging from the same day to 56 days). The number of nonattending patients according to their waiting time in days until the consultation is presented in Fig. 2.

The largest number of respondents who did not attend the planned consultations were those who waited for consultation for 7 days (7.5%).

A total of 375 respondents (96.4%) identified 13 reasons for nonattendance, while 14 respondents (3.6%) could not identify any reason for nonattendance. The reasons for not attending consultations and the mean waiting time until the planned outpatient consultations are presented in Table 2.

**Table 2:** The main reasons for not attending planned outpatient consultations and the mean waiting time according to the reasons for nonattendance

Reasons for nonattendance	Respondents(n)	Proportion (%)	Mean waiting time (days) ± SD
Worsened health status	96	24.69	14.48 ± 9.56
Unidentified personal problems	58	14.91	14.31 ± 9.41
Work-related problems	53	13.62	16.56 ± 10.68
Forgot about the planned consultation	46	11.82	14.17 ± 9.75
Health problem was solved at another health care institution	34	8.74	18.37 ± 10.45
Did not have/get a referral to the consultation	19	4.9	11.26 ± 10.61
Recovered	17	4.37	11.71 ± 5.30
Unknown reason for nonattendance (responder could not identify any reason)	14	3.6	17.50 ± 7.03
Too long of a waiting time until the planned consultation	12	3.08	20.58 ± 9.46
Changed his or her mind	12	3.1	16.75 ± 17.77
Family member's illness	10	2.6	14.5 ± 9.60
Transportation problems	8	2.1	16.25 ± 9.33
Was registered with another specialist at the same time	6	1.5	9.67 ± 12.50
Death of a family member	4	1.0	13.25 ± 6.94

The most common reasons for nonattendance were worsened health status (24.69%), personal problems (14.91%), and work-related problems (13.62%). The longest average waiting time until the consultation was for the patients who reported “too long of a waiting time until the planned consultation” (20.58 ± 9.46 days) as a reason for nonattendance. The shortest average waiting time until the consultation was for the patients who “registered with another specialist at the same time” (9.67 ± 12.50 days).

The mean waiting time of the respondents for a new (primary) consultation was 15.68 ± 9.5 days, while the mean waiting time for a follow-up (secondary) consultation was 11.23 ± 10.3 days. A significant difference was found when comparing the means of the waiting times according to appointment type (*p* < 0.05).

Nonattendance occurred for 19 specialty areas. Respondents who registered for a planned consultation most often did not attend appointments with neurologists (17.0%, mean waiting time was 15.62 ± 12.34 days), traumatologists (11.3%, mean waiting time of 13.36 ± 7.23 days) and cardiologists (10.5%, mean waiting time of 13.49 ± 9.95 days). The longest average waiting time was observed for the respondents who had registered for consultations with gastroenterologists (26.0 ± 6.99 days), while the respondents who were registered for consultations with gynaecologists (4.13 ± 2.99 days) had to wait for the shortest amount of time. The list of all specialists as well as the mean waiting time until the planned outpatient consultation according to each specialist type are represented in Table 3.

**Table 3:** Distribution of nonattending respondents by specialist and average waiting time

Specialist	Respondents (n)	Proportion (%)	Mean waiting time (days) ± SD
Neurologist	66	17.0	15.62 ± 12.34
Traumatologist	44	11.3	13.36 ± 7.23
Cardiologist	41	10.5	13.49 ± 9.95
Otorhinolaryngologist	38	9.8	10.16 ± 6.83
Ophthalmologist	36	9.3	14.67 ± 9.03
Pulmonologist	32	8.2	16.06 ± 8.35
Vascular surgeon	32	8.2	25.53 ± 9.95
Rehabilitation physician	22	5.7	19.55 ± 5.06
Endocrinologist	17	4.4	14.53 ± 9.61
Rheumatologist	16	4.1	15.44 ± 10.99
Gastroenterologist	10	2.6	26.0 ± 6.99
Urologist	9	2.3	6.0 ± 2.71
Gynaecologist	8	2.1	4.13 ± 2.99
Surgeon	6	1.5	7.5 ± 4.41
Clinical physiologist	5	1.3	15.0 ± 7.21
Allergologist	2	0.5	15.5 ± 2.12
Neurosurgeon	2	0.5	12.0 ± 8.48
Nutritionist	1	0.3	11.0
Internal medicine specialist	1	0.3	5.0

Using multinomial logistic regression analysis, the overall contribution of waiting time, sex and age of the patients, as well as the type of planned consultation (primary or follow-up), to each reason for nonattendance was tested. In each model, the Nagelkerke pseudo-R squared value was > 0.25. A longer waiting time was significantly associated with the following reasons for nonattendance: work-related problems (χ^2^ = 62.55; *P* = 0.034), health problems solved at another health care institution (χ^2^ = 86.15; *P* < 0.001), unidentified personal problems (χ^2^ = 67.9; *P* = 0.012) and unknown reasons for nonattendance (χ^2^ = 64.97; *P* = 0.022). The statistical significance of all variable contributions to each reason for nonattendance is presented in Table 4.

**Table 4:** Multinomial logistic regression analysis of variables associated with the reasons for nonattendance

Reasons for nonattendance	Factors, *P* values			
	Longer waiting time	Sex	Age	Appointment type
			(Older patients)	(New referral)
Worsened health status	> 0.05	> 0.05	** < 0.001**	> 0.05
Unidentified personal problems	**0.012**	> 0.05	**0.008**	** < 0.001**
Work-related problems	**0.034**	> 0.05	> 0.05	> 0.05
Forgot about the planned consultation	> 0.05	> 0.05	> 0.05	**0.028**
Health problem was solved at another health care institution	** < 0.001**	> 0.05	**0.034**	**0.01**
Did not have/get a referral for the consultation	> 0.05	> 0.05	> 0.05	> 0.05
Recovery	> 0.05	> 0.05	> 0.05	> 0.05
Unknown reason for nonattendance (responder could not identify any reason)	> 0.05	> 0.05	> 0.05	> 0.05
Too long of a waiting time until the planned consultation	> 0.05	> 0.05	> 0.05	> 0.05
Changed his or her mind	> 0.05	> 0.05	**0.007**	** < 0.001**
Family member's illness	> 0.05	> 0.05	> 0.05	> 0.05
Transportation problems	> 0.05	> 0.05	> 0.05	> 0.05
Was registered with another specialist at the same time	> 0.05	> 0.05	> 0.05	> 0.05
Death of a family member	> 0.05	> 0.05	> 0.05	> 0.05

The appointment type was a very important predictor of particular reasons for nonattendance. A new referral was significantly associated with the following reasons: unidentified personal problems (χ^2^ = 13.39; *P* < 0.001), being unaware of the planned consultation (χ^2^ = 4.58; *P* = 0.028), health problems were solved at another health care institution (χ^2^ = 6.57; *P* = 0.01), and the patient changed their mind (χ^2^ = 16.32; *P* < 0.001). Older age was significantly associated with the following reasons: worsened health status (χ^2^ = 38.33; *P* < 0.001), unidentified personal problems (χ^2^ = 99.15; *P* = 0.008), health problems solved at another health care institution (χ^2^ = 90.82; *P* = 0.034) and the patient changed their mind (χ^2^ = 100.35; *P* = 0.007). Sex was not identified as an important factor for any of the reasons for nonattendance.

## Discussion

The mean waiting time until the planned outpatient consultation was 15.13 ± 10 days in our study. A total of 7.5% of the patients did not attend the consultations when the waiting time was several days, and for 6.5% of patients, the waiting time was 15 days. The highest proportion for nonattendance was observed when the wait time was between 6 and 17 days (59.6% of the total number of nonattending patients). McIntyre D and Chow CK summarized waiting time to see a specialist in different countries and concluded that waiting time to specialist access is high and increasing in some OECD countries. In Canada, specialist waiting time was 8.7 weeks in 2017; in Australia, the median wait time, calculated by combining reported medians across all specialties and centres, was 5.9 months in 2019. A study conducted in 2004 across European countries found a wide intercountry variation in self-reported data, from 0.86 weeks in Greece to 9.65 weeks in Sweden [16]. A guide to NHS waiting times in England reports that the maximum waiting time for nonurgent, consultant-led treatments is 18 weeks from the day the appointment is booked or when the hospital or service receives your referral letter [17]. Thus, the waiting time estimated in our study is very low compared with that in other countries.

The results of our study cannot confirm the statement that increasing waiting times cause higher nonattendance rates. In fact, nonattendance was the highest when waiting times were moderate: from 6 to 17 days, when 59.7% of patients missed their consultations. In addition, even same-day consultations had a 4.4% nonattendance rate. Our results are similar to those of Mohammadi I et al., who found that the highest rate of nonattendance was 35.4% within two weeks and 20.7% for more than two weeks in community health centres [9]. In contrast to our findings, Cohen A stated that as waiting times increase, the frequency of nonattendance increases. The author concluded that with a waiting time of 1 to 7 days, the nonattendance rate is 21.2%; with a waiting time of 8 to 14 days, the nonattendance rate is 32.5%; and waiting times longer than 15 days increase the nonattendance rate to 43.5% [7].

The first hypothesis, which was that one of the prevailing reasons for nonattendance is a waiting time that is too long, was rejected according to the results of this study. The highest proportion for nonattendance to the planned outpatient consultation was worsened health status (24.69%, mean waiting time until consultation 14.48 ± 9.56 days), unidentified personal problems (14.91%, mean waiting time until consultation 14.31 ± 9.41 days) and work-related problems (13.62%, mean waiting time until consultation 16.56 ± 10.68 days). It should be noted that only 3.08% of the respondents identified the reason for nonattendance as “too long of a waiting time until the planned consultation”, with the longest mean waiting time being 20.58 ± 9.46 days in our study.

In general, the reasons for nonattendance that were identified in our study are similar to other studies, but their frequencies are different. According to Neal RD et al., forgetfulness is the main cause for nonattendance, with over 40% of patients reporting this as the reason for missing the consultation [10]. Similarly, Shahab I and Meili R, in a study of nonattendance in a community clinic, found that 32.6% of the respondents forgot about their appointments, with the second most common reason being declining health status (23.3%) [18]. Vaeggemose U et al., in a study of nonattendance in public hospitals, concluded that 18% of the respondents reported forgetfulness [19]. A total of 11.82% of the patients forgot about the appointments (mean waiting time until consultation was 14.17 ± 9.75 days) in our study. The audit of the National Audit Office in Lithuania has revealed that long queues are also partially caused by the patients themselves, as approximately 20 percent of those who have secured an appointment at a medical establishment fail to arrive [6]. Our study showed that the rate of nonattendance was 4.49% in Kaunas Hospital. The lower rate of nonattendance could be explained by specific measures taken at Kaunas Hospital, such as reminders of the consultation date and time by SMS and/or control over registered planned outpatient consultations by several outpatient specialists at the same time within Kaunas Hospital.

The association between physician specialty and attendance varies widely. A study by Kheirkhah P et al. found that the highest rates of nonattendance in the studied health centres were for consultations with gastroenterologists and otorhinolaryngologists [2]. Dantas LF et al. concluded that most patients did not attend consultations with psychiatrists [15]. In our outpatient clinic, nonattendance was the highest among patients visiting neurologists (17%), traumatologists (11.3%) and cardiologists (10.5%). However, contrary to the previously mentioned studies, gastroenterologists had the lowest nonattendance rates (4.1%). Heterogeneity of the results shows that nonattendance is context sensitive, and many unmeasured factors play a role in outpatient clinics that can be addressed only individually; for example, health care institutions of different profiles do not employ all types of medical professionals.

In our study, we emphasized the waiting time aspect, but the results did not confirm the second hypothesis, which stated that a longer time between referral and appointment is associated with higher rates of nonattendance. In our study, a longer waiting time was significantly associated with the following reasons for nonattendance: work-related problems, health problems solved at another health care institution, unidentified personal problems and unknown reasons for nonattendance. A longer waiting time was not significantly associated with the reason “too long of a waiting time until the planned consultation”. Our study showed that the issue of waiting time until the planned consultation is not an important aspect of nonattendance, which is in contrast to the statements of the National Audit Office in Lithuania.

In general, the results of the study show that a few administrative actions can be taken to decrease the number of nonattending patients. Out of 14 identified reasons for nonattendance, the majority of them can be treated as subjective reasons, with no possibility of influence by providers of health care services. One of the identified reasons— “forgot about the planned consultation”—can be influenced by the health care provider by increasing the number of SMS reminders about the time of the planned outpatient consultation. Another identified reason for nonattendance—the patient “did not have/get a referral for the consultation”—could be managed by asking the family physician to provide information about acquiring a referral. The reason “was registered with another specialist at the same time” could be managed by prohibiting registration with another specialist, at least at the same time, in the same health care institution.

This study has several limitations. We only analysed nonattending patients and did not analyse patients who attended the planned outpatient consultation. We only focused on nonattending patients and designed the study to answer one main question—how does the waiting time until the planned outpatient consultation influences the reason for nonattendance? Another limitation of this study is that we were unable to contact nonattending patients who did not have regular access to telephones or who might have been unavailable during call hours. Due to this, there could be some deviations from the sociodemographic characteristics of the nonattending patients, as well as the proportions of the reasons for nonattendance. In predicting this situation, we calculated a sample size that could represent the population of nonattending patients. The short waiting time until the planned outpatient consultation (mean 15.13 ± 10 days; range from nil to 56 days) could also be considered a limitation of this study. A truncation effect could have an impact on exploring the association between waiting time and nonattendance.

## Conclusions

The mean respondents’ waiting time until the planned outpatient consultations was 15.13 ± 10 days. The highest proportion of nonattendance was observed when the wait time was between 6 and 17 days. The patients did not identify the long waiting time until outpatient specialist consultation among the main reasons for nonattendance. They more often did not attend the planned outpatient consultation due to worsened health status (24.69%, mean waiting time until consultation 14.48 ± 9.56 days), unidentified personal problems (14.91%, mean waiting time until consultation 14.31 ± 9.41), or work-related problems (13.62%, mean waiting time until consultation 16.56 ± 10.68 days). A longer waiting time was significantly associated with the following reasons for nonattendance: work-related problems, health problems solved at another health care institution, unidentified personal problems and unknown reasons for nonattendance. The highest proportions of nonattending patients were registered with neurologists (17.0%, mean waiting time was 15.62 ± 12.34 days), traumatologists (11.3%, mean waiting time was 13.36 ± 7.23 days) and cardiologists (10.5%, mean waiting time was 13.49 ± 9.95 days). The issue of waiting time until the planned consultation is not an important aspect of nonattendance.

## Data Availability

All data generated or analysed during this study are included in this published article.
